# Percutaneous hollow nail internal fixation treatment for fractures of the pubic symphysis and its adjacent areas

**DOI:** 10.3389/fsurg.2024.1400834

**Published:** 2024-10-29

**Authors:** Zhang Ping

**Affiliations:** Department of Orthopedics, Zibo Municipal Hospital, Zibo, China

**Keywords:** pubic symphysis, fracture, percutaneous, hollow nail, pain

## Abstract

**Purpose:**

To explore the percutaneous minimally invasive treatment of pubic symphysis and its adjacent fractures.

**Method:**

Since May 2021, 13 cases of fractures involving the pubic symphysis and its adjacent parts were treated with x-ray fluoroscopic localization and percutaneous cannulated screw fixation across the symphysis pubis, the guide pin pierced the symphysis pubis and the fracture end and stopped at the inner edge of the acetabulum. Visual analogue scale (VAS) was used to evaluate the effect of the operation, and the patients were followed up.

**Result:**

The screw insertion operation time was 25–40 min, with an average of 31.45 min; The number of perspectives is 20–40, with an average of 28.75. The average intraoperative blood loss was 10 ml, and there were no puncture complications such as nerve or vascular damage. The initial stability of the fractured end of the patient after surgery was good. The VAS score decreased from preoperative 8–10 points to postoperative 1–2 points (average of 1.5 points). The follow-up time was 3–25 months, with an average of 8.5 months. At the last follow-up, the excellent and good rate of pelvic function according to Majeed pelvic function scoring system was 100%. One patient had screws removed 1.5 years after surgery, while the remaining twelve patients did not have screws removed. All patients did not experience any discomfort symptoms caused by pubic symphysis fixation.

**Conclusion:**

Percutaneous hollow nail internal fixation is an effective method for treating fractures of the pubic symphysis and its adjacent parts.

## 1 Introduction

Percutaneous cannulated screw fixation is an ideal method for the treatment of anterior pelvic ring fractures. It is often used for fractures involving the middle and lateral acetabular branches of the superior pubic bone (1–4). The symphysis pubis and its adjacent parts are the traditional pubic ramus screw entry point or the head anchor position, which is difficult to be fixed with the traditional pubic ramus screw after fracture. In order to increase the span and anchoring strength of the screw, percutaneous hollow screw fixation was performed through the symphysis pubis of the contralateral side in the study. After preliminary attempts, ideal clinical results were achieved, as reported below.

## 2 Surgical techniques

### 2.1 Preoperative preparation

**Figure 1:**
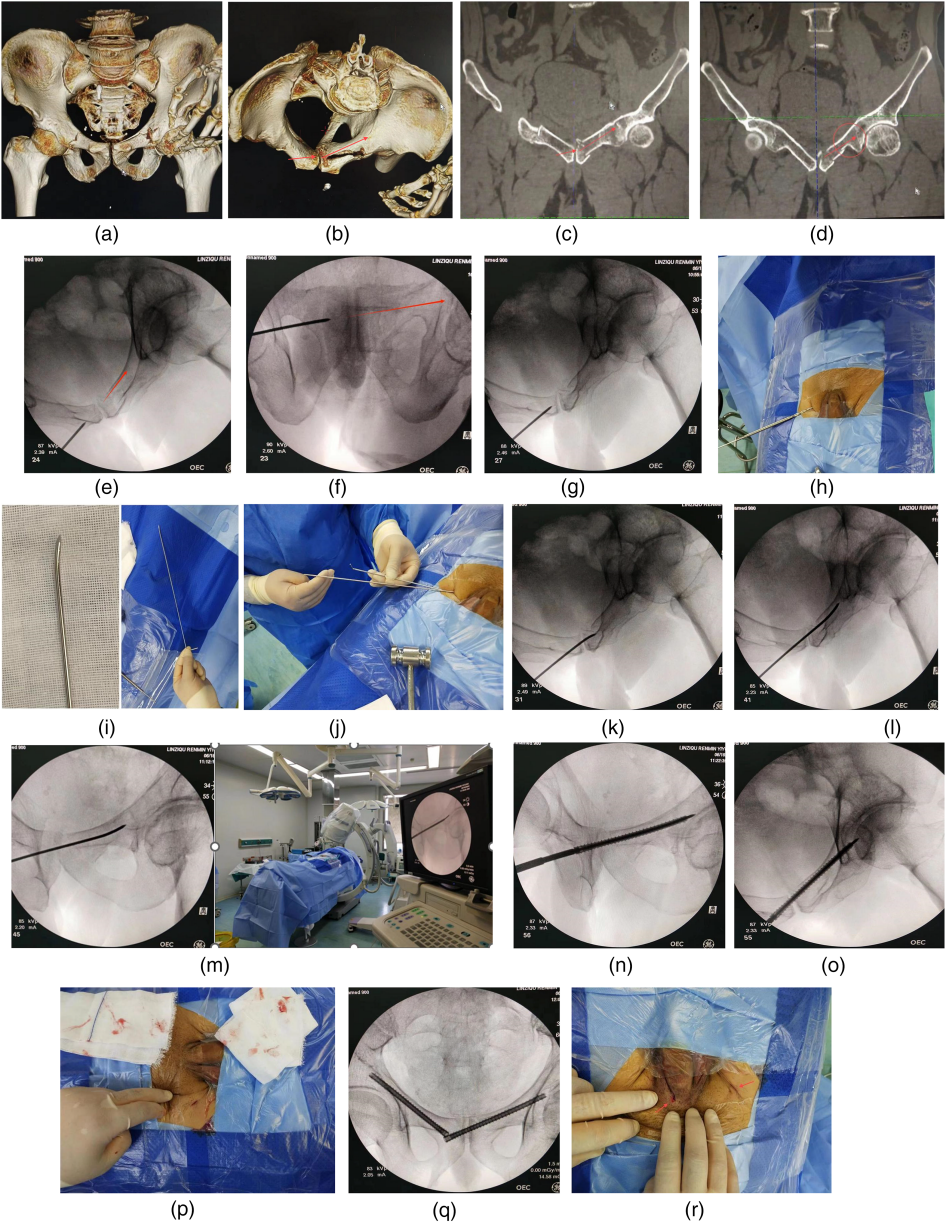
**(a)** Pelvic fractures included both pubic branches and left sacral wing. **(b)** The left superior pubic branch split laterally and involves the symphysis pubis. The fixation position was designed and labeled by the arrow. **(c)** The feasibility of transpubic symphysis screw fixation was confirmed and the puncture path was planned by coronal CT scan. **(d)** It was clear that there was a relatively intact anchoring area at the acetabular fracture end, which was largely surrounded by three sides of cortical bone. **(e)** Pelvic inlet fluoroscopy: The extension line of the guide pin was close to the posterior cortex of the pubic branch on the affected side. **(f)** Pelvic outlet fluoroscopy: The extension line of the guide pin was located between the upper margin of the obturator foramen and the central axis of the pubis designed for this patient. **(g)** Hammered the guide pin into the bone about 1 cm. **(h)** Used a hollow drill to enlarge the bone inlet. **(i)** Took another guide pin of the same size and shaped it for use. **(j)** Replaced the guide pin after exiting the hollow drill. **(k)** Both sides of the pubic symphysis were penetrated with shaped guide pin. **(l)** Adjusted the direction of the guide pin by adjusting the direction of the guide pin end to follow the posterior margin of the pubic branch. **(m)** Adjusted the perspective direction to ensure that the path was long enough without interfering with the acetabulum. **(n)** A hollow screw of 6.5 mm diameter was nailed along the guide pin. **(o)** The fracture alignment was well observed by pelvic inlet fluoroscopy. **(p)** Incision size, appearance and total blood loss (gauze impregnation). **(q)** The contralateral pubic rami were subsequently fixed with retrograde hollow nail. **(r)** The incision appearance of both screws with one of the incisions was already closed.

Preoperative pelvic x-ray, CT scan and three-dimensional reconstruction were performed to determine the shape of fracture and the direction of displacement, to evaluate the feasibility of percutaneous cannulated screw fixation and make a surgical plan. Pre-design of screw placement: CT coronal scan was used to pre-design the best puncture site and path (Figure 1c), in order to ensure that the screw head could enter the affected side pubic branch, the best choice was to run the screw through the healthy bone at most. The location of the puncture point relative to the pubic tubercle could be judged by comparing the corresponding relationship between the level and the scanning layers of the superior margin of pubic symphysis and the inferior angle of pubic symphysis, the puncture point was generally at the level of the middle and upper 1/3 junction of the symphysis pubis just below the pubic tubercle.

### 2.2 Anesthesia and positioning

Local infiltration anesthesia with intravenous assistance or general anesthesia, supine position.

### 2.3 Operation procedure shown by a case

#### 2.3.1 Patient information

This patient was a 53-year-old male with a fall injury from a height and a fracture of the left superior rami pubis involving the symphysis pubis (Figures 1a,b). Surgery was performed 3 days after the injury. In order to increase screw length due to the extended fracture area span, non-standard obturator exit perspective was added in addition to entrance and exit perspective of pelvis. The operations are performed under non-x-ray exposure for the surgeon. Each perspective used a single exposure rather than a continuous perspective. And the operation was performed under general anesthesia.

#### 2.3.2 Preoperative surgical design

Figures 1b–d.

#### 2.3.3 Operation procedure

A 1 cm incision was made at the lower inguinal skin fold of the contralateral side after 1:200,000 parainrenin solution was infiltrated. Vascular forcep was inserted to penetrate the pubic muscle membrane and directly reached the lateral bone of the pubic symphysis, and blunt dissection was performed to establish a soft tissue channel. Then, a 2.5 mm diameter and 300 mm length steel bone pin as a guide pin was replaced for puncture under the guidance of pelvic inlet and outlet fluoroscopy (Figures 1e,f). After defining the puncture path and entry point which was particularly importent, the cortical bone was penetrated at the entry point and penetrated about 1 cm (Figure 1g). The entry point and guide pin orientation were confirmed by x-ray fluoroscopy again. If the orientation of the guide pin completely conformed to the design path, the guide pin should further penetrate the symphysis pubis and pass through the fracture end to the contralateral healthy bone. Otherwise, taken the following measures to continue the pin insertion operation. Used a hollow drill to widen the puncture inlet along the guide pin (Figure 1h). Another guide pin was prebent and shaped so that the tail end was easy to handle and the head end was easy to change the direction of intramedullary puncture, and the direction of the head end could be judged by the direction of the tail end through the above design (Figure 1i). Withdrawn the hollow bit without moving the inner pin, and placed the pre-bent guide pin along the unshaped guide pin into the enlarged bone entrance, then remove the unshaped guide pin (Figure 1j). Under fluoroscopy, held the shaped guide pin and deepen along the designed path through the pubic symphysis and through the fracture end till to the contralateral healthy bone (Figures 1k–m). After straighting the bent end of the pin and comparing with the same size guide pin to measure the penetration depth, a 6.5 mm fully threaded hollow nail (Watson) was screwed along the guide pin for fixation after the end of the pin was cut of (Figures 1n,o). Then bent the guide pin again and pulled it out with force. A guide pin with a diameter of 2.3 or 2.4 mm should be more useful for puncture, which is easier to remove if it can be obtained. It is not easy to control the direction of the head during deep puncture with a guide pin less than or equal to 2 mm diameter.

### 2.4 Postoperative treatment

Active flexion and extension of hip joint, turning activities in bed and semi-sitting were encouraged after conscious anesthesia. After 2–3 days, the patients were able to stand on the ground with both crutches according to the general condition. After 1 month, partial weight-bearing activities were permeted with the help of a single crutche. After 2 months, full weight bearing activities were performed. Visual analogue scale (VAS) was used to evaluate the early effect of surgery. The VAS score was mainly based on the maximum pain associated with pelvic fracture induced by the patient's rolling over and hip flexion and extension, rather than the pain at the pubic fracture site alone. Scoring began for patients under general anesthesia after the anesthetic wore off, usually on the second day after surgery. Scoring for patients under local anesthesia patients were scored after surgery, usually in the operating room. All patients were scored once a day from the second day after surgery, and continuously scored until discharge. The quality of fracture reduction and the position of internal fixation were evaluated according to the Matta score (5): fracture displacement <4 mm was considered excellent, 5–10 mm was considered good, 11–20 mm was considered acceptable, and >20 mm was considered poor. The patients were followed up and x-ray films were taken 6 and 12 weeks after operation. At the last follow-up, the curative effect was evaluated by Majeed pelvic function scoring system (6): work, pain, sitting, standing and sexual life were scored, the full score was 100, ≥85 was excellent, 70–84 was good, 55–69 is fair, <55 is poor.

## 3 Clinical data

### 3.1 General information

There were 13 patients in this group, 5 male and 8 female, aged from 27 to 72, with an average of 53.5 years old. All were TileB type pelvic fractures involving the pubic symphysis. The contralateral symphysis pubis had intact bones, and one side of the alar sacrum was fractured but vertically stable. There were 5 cases of traffic accident, 3 cases of high fall injury, 2 cases associated with falls while riding electric bicycle, 1 case of crush injury, 2 case of low energy fall injury. Among them, 3 cases were complicated with open fracture of tibia and fibula, fracture of femoral shaft, multiple rib and lumbar vertebral fracture respectively. One patient with a fall injury sought medical attention and underwent surgery 2 months after injury, while the rest of the injuries took 2–10 days until surgery, with an average of 3.75 days. This study had been approved by the hospital ethics committee and all patients had informed consent.

### 3.2 Preliminary results

The screw insertion operation time was 25–40 min, with an average of 31.45 min; The number of perspectives is 20–40, with an average of 28.75. The average intraoperative blood loss was 10 ml, and there were no puncture complications such as nerve or vascular damage. The initial stability of the fractured end of the patient after surgery was good. The VAS score decreased from preoperative 8–10 points to postoperative 1–2 points (average of 1.5 points). The follow-up time was 3–25 months, with an average of 8.5 months. All fresh fractures healed at a follow-up of 3 months after surgery. One patient had screws removed 1.5 years after surgery, while the remaining 12 patients did not have screws removed. All patients did not experience any discomfort symptoms caused by pubic symphysis fixation. At the last follow-up, the excellent and good rate of pelvic function was 100% according to the Majeed pelvic function scoring system.

#### 3.2.1 Typical cases

##### 3.2.1.1 Case 2

**Figure 2:**
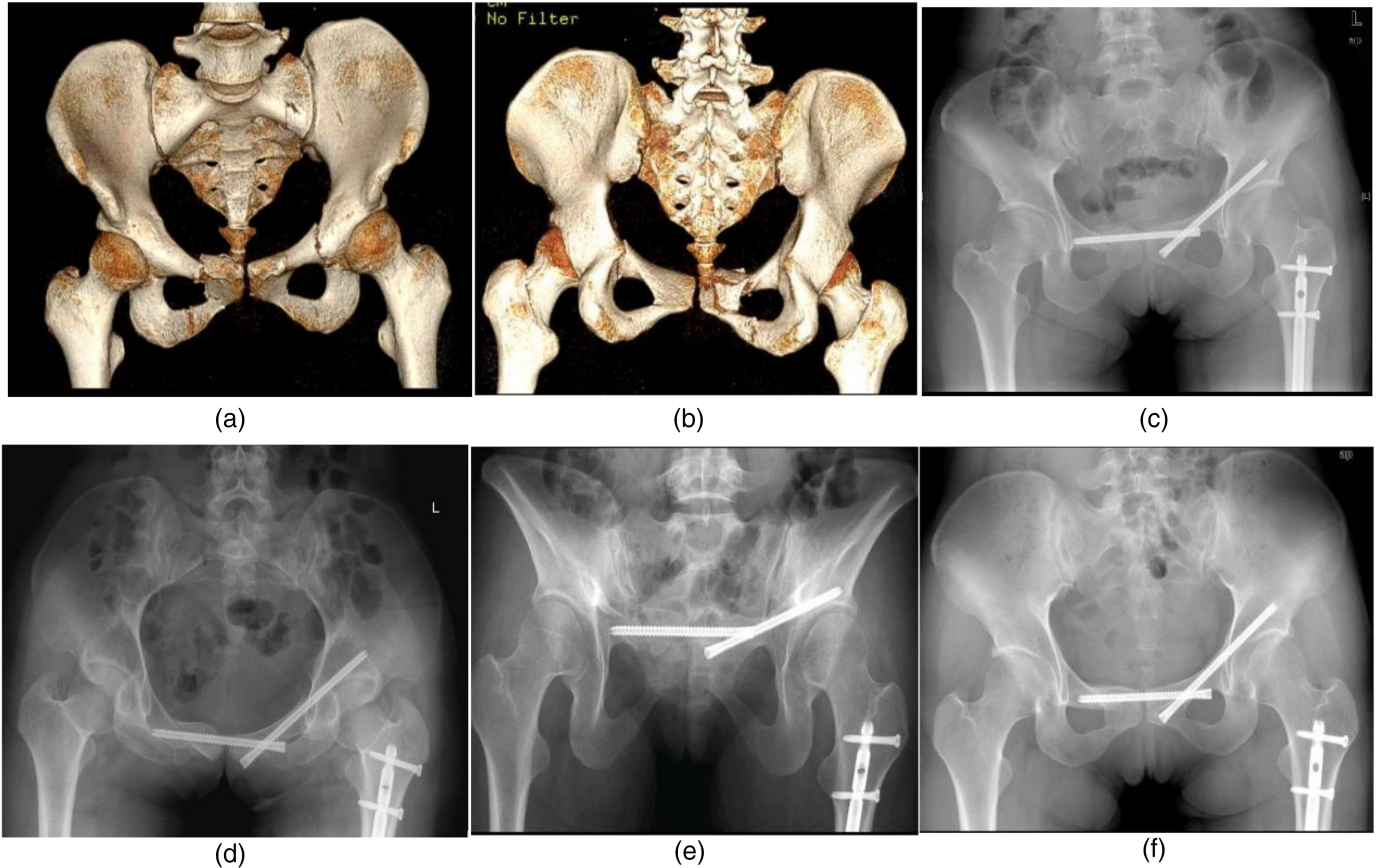
**(a)** Preoperative 3D CT anterior view: Right pubic symphysis fracture, combined with left sacral wing and superior pubic branch acetabular side fracture. **(b)** Preoperative 3D CT posterior view: The integrity of symphysis pubis on the right side of posterior view was obviously damaged. **(c)** Screw position as shown in the postoperative anteroposterior view of the pelvis. **(d)** Screw position as shown in the postoperative pelvic entrance view. **(e)** The screw position as shown in the postoperative pelvic outlet view. **(f)** The anteroposterior view of the pelvis at 6 months after operation showed good fracture healing and no loosening of the screws.

Female, 27 years old, injured in a car accident. Right pubic symphysis fracture, combined with left sacral wing and superior pubic branch acetabular side fracture, preoperative VAS score for turning activity was 9 points, and postoperative VAS score decreased to 1 point after percutaneous screw fixation. Figures 2a,b: Preoperative CT images. Figures 2c–e: Postoperative images of the entrance and exit positions. Figure 2f: Six months after the operation, the fracture healed well.

##### 3.2.1.2 Case 3

**Figure 3:**
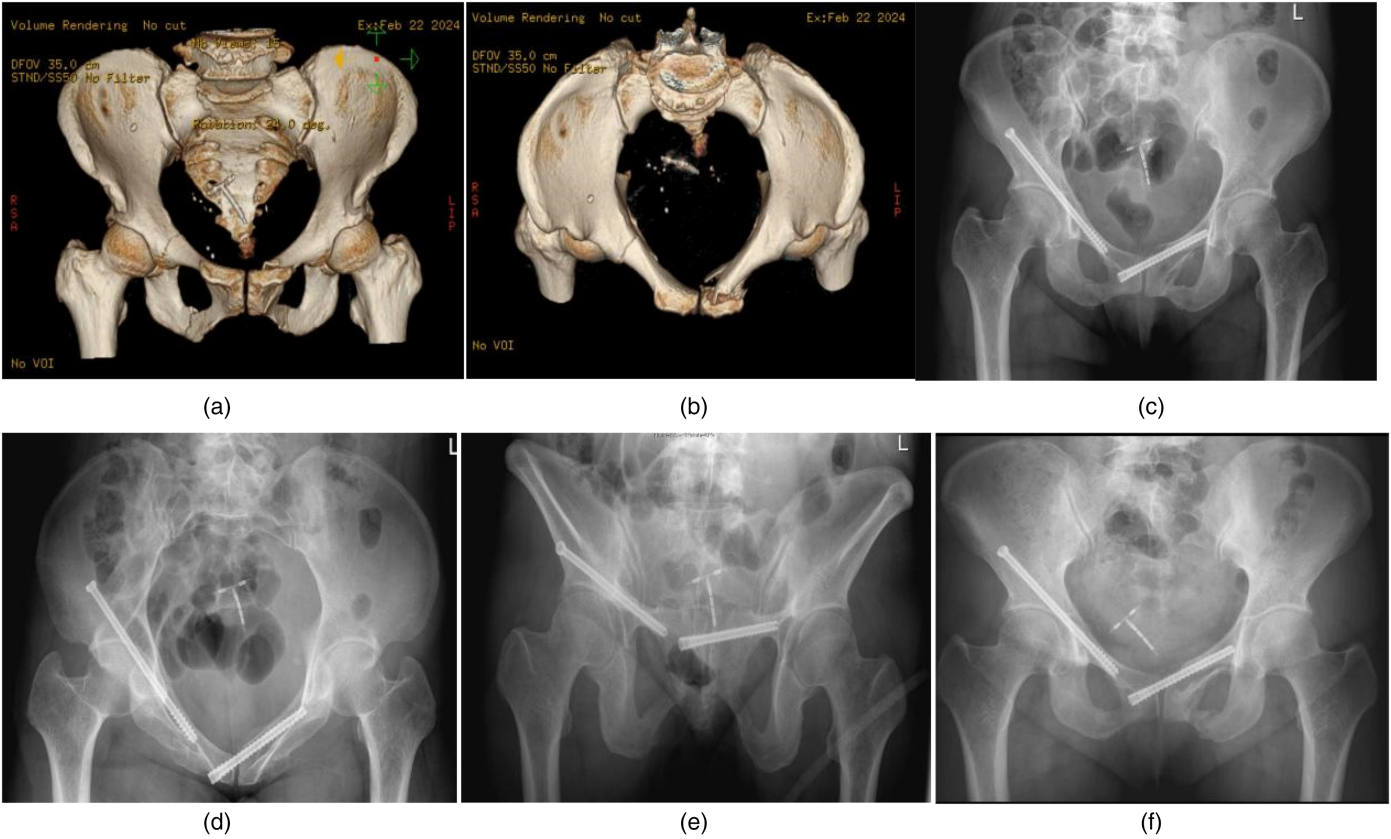
**(a)** Preoperative 3D CT anterior view: Bilateral anterior pelvic ring fractures including left symphysis pubis fracture. **(b)** Preoperative 3D CT entrance view: The integrity of the pubic symphysis was destroyed. **(c)** Screw position as shown in postoperative pelvic anteroposterior view. **(d)** Screw position as shown in the postoperative pelvic entrance view. **(e)** The screw position as shown in the postoperative pelvic outlet view. **(f)** Anteroposterior pelvic images at 3 months postoperatively showed callus formation and no screw loosening.

Female, 51 years old, with crush injury. Left pubic symphysis fracture, combined with right sacral region II and superior pubic branch acetabular side fracture, preoperative VAS score for turning activity was 10 points, and after percutaneous internal fixation, VAS score decreased to 1 point. Figures 3a,b: Preoperative CT images. Figures 3c–e: Postoperative pelvic x-ray, inlet x-ray, outlet x-ray. Figure 3f: The fracture healed well 3 months after operation.

##### 3.2.1.3 Case 4

**Figure 4:**
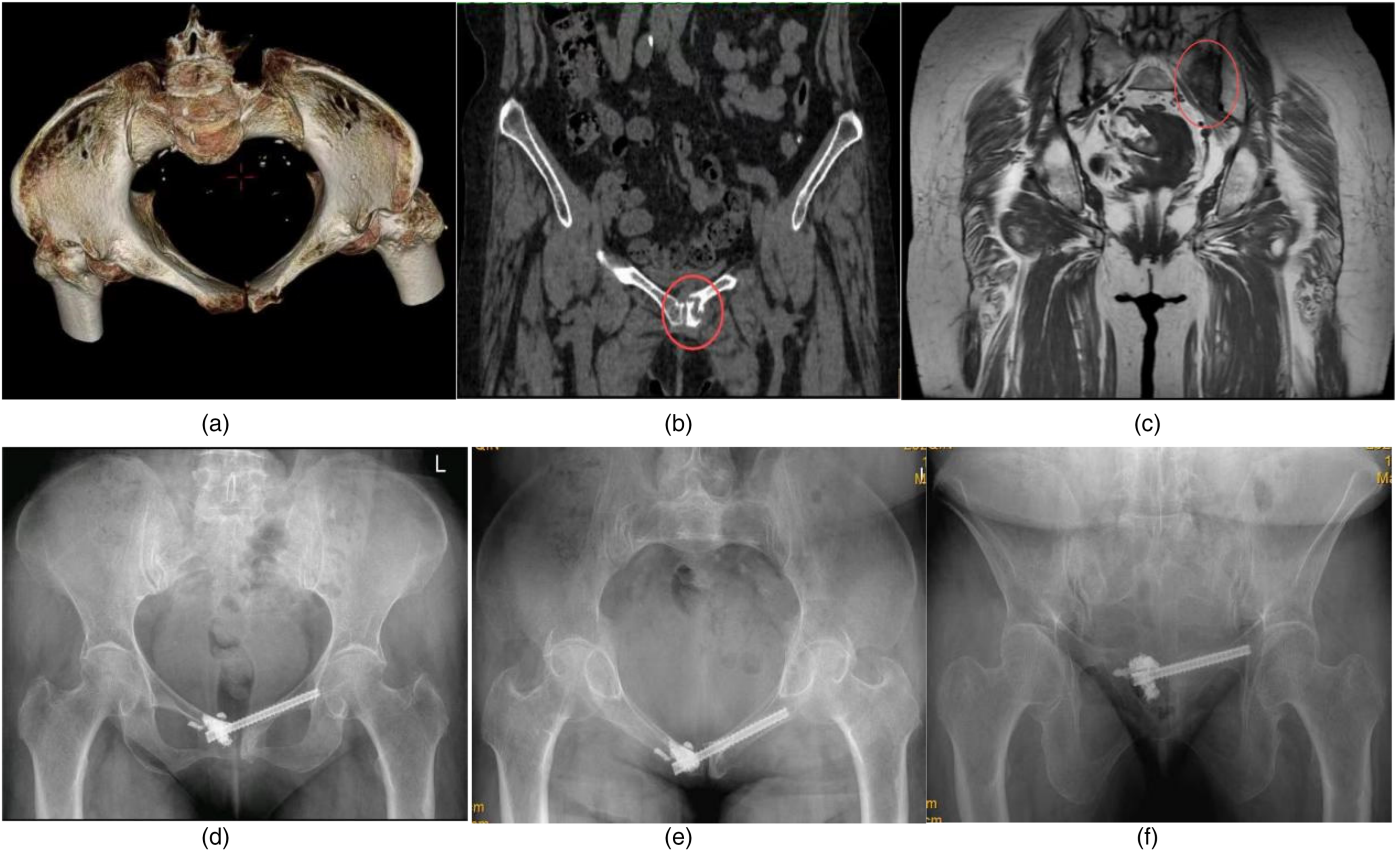
**(a)** Preoperative CT entrance view showed a fracture of the left pubic symphysis adjacent to the articular surface. **(b)** Preoperative CT scan showed sclerosis and nonunion of the symphysis pubis fracture. **(c)** Preoperative MR Showed that ipsilateral sacral alar bone marrow edema. **(d)** Postoperative anteroposterior pelvic images: cannulated screw fixation was performed across the pubic symphysis and bone cement reinforcement was performed at the end of the screw. **(e)** Screw position as shown in the postoperative pelvic entrance view. **(f)** The screw position as shown in the postoperative pelvic outlet view.

Female, 62 years old. Concomitant rheumatoid arthritis and osteoporosis. Fall causing pubic symphysis fracture combined with left sacral wing fracture. Two months after the injury, the patient sought medical attention and had a preoperative VAS score of 8.5 for hip pain during walking and turning activities in bed, and a VAS score of 2 for pain at the site of the pubic symphysis fracture. Percutaneous hollow nail internal fixation and tail end bone cement enhancement resulted in a decrease in hip VAS score to 2 points. At the 6th week of follow-up, the pain symptoms disappeared, but the VAS score of the left groin area was 1 point when walking more than 3,000 steps, and there was no change in the follow-up for 6 months. Figures 4a,b: Preoperative CT shows non union and hardening of the fracture end. Figure 4c: Preoperative magnetic resonance imaging revealed edema signals on the left sacral wing. Figures 4d–f: Postoperative pelvic x-ray, inlet x-ray, outlet x-ray.

## 4 Discussion

TileB type pelvic fractures were common in clinical practice, and most fractures were not seriously displaced. Fractures healing could be achieved in most young patients after conservative treatment, but it required prolonged bed braking, while Hoch et al. reported that the reoperation rate of elderly patients with lateral compression pelvic fractures after conservative treatment failure was as high as 18% (7). After a pelvic ring fracture, turning over, coughing, and defecation could cause severe pain, often accompanied by limited active hip flexion and extension, increasing nursing difficulty and bed complications. In some elderly patients, the pain aggravated rather than alleviated after conservative treatment. The main cause might be the abnormal post-structural stress caused by unstable pelvic rotation, resulting in changes in sacral bone marrow pressure, periostosis or iliolumbar fascia tension. Therefore, after the failure of conservative treatment, people often went to the hospital for serious hip pain which could be alleviated by stabilizing the anterior pelvic ring in our experience. Slight rotation and displacement after pubic symphysis fracture could lead to fracture end separation, and the fracture alignment seen in imaging could not reflect the fracture alignment of patients when they were active. Stress tests under anesthesia had been therefore recommended to detect latent unstable pelvic fractures (8–10).

Due to factors such as thin bone and osteoporosis, the failure rate of conservative treatment or indirect fixation with external fixator was relatively high according to our experience. A prospective controlled study showed that patients with slightly displaced lateral compression fractures benefited more from surgery than from conservative treatment (11). Another retrospective, multicenter study showed that loss of walking ability was associated with reduced life expectancy in patients treated conservatively (12). Therefore, for this type of fracture, we advocate active minimally invasive direct internal fixation rather than conservative treatment or external fixation or INFIX indirect fixation because even micro movement at the fracture site could lead to persistent dull pain, limited hip mobility, and difficulty in achieving rapid recovery (13–15). Additional reasons why external pelvic fixators are not recommended include nail path infection, limited mobility, nerve compression, and soft tissue stimulation (16, 17). While reasons for INFIX not being recommended include lateral femoral cutaneous nerve injury, femoral nerve injury, poor patient comfort, external iliac artery compression, bladder incarceration and other complications (18–21). For patients with osteoporosis, high non-fatal complications caused by open surgery which was highly controversial had been reported in the literature (22). Therefore, open surgery which need to sever the insertion of the rectus abdominis muscle is also not the preferred treatment option.

Percutaneous hollow nail internal fixation is an ideal surgical method for treating pubic ramus fractures (23–25), but it is mainly used for zone II and III fractures in Nakatani classification (26). The pubic symphysis is located in zone I, and compared to zones II and III, the bone in this area is thin and mainly composed of spongy bone. When the fracture involves this area, traditional pubic screw fixation anchor is difficult, lacking a biomechanical stable anatomical basis, and the short-term surgical effect is difficult to ensure. Therefore, for the surgical treatment of Nakatani type I fractures, current literature reports mainly focus on open surgery or small incision steel plate internal fixation (27–29), which is not minimally invasive compared to percutaneous hollow nail internal fixation. Cross pubic symphysis fixation could utilize the intact bone of the contralateral pubic symphysis, especially the subchondral bone of the bilateral pubic symphysis joint, to increase the strength of the screw anchor. The pubic symphysis is a micro moving joint attached by strong ligaments, which could only be slightly displaced vertically and rotated horizontally under normal circumstances (30, 31). Therefore, its fixation effect is relatively small. This study followed up 13 patients for 3–25 months, one of whom experienced full-term pregnancy 1 year after surgery without removing the screw. All patients did not experience any discomfort symptoms caused by pubic symphysis fixation.

It is worth noting that although this study has theoretical advantages in minimally invasive and accelerated rehabilitation, comprehensive preoperative evaluation and nail placement planning are necessary for clinical application. Patients with hip extension disorders on the contralateral side should be selected carefully. Obstruction of the thigh may have a certain impact on nail placement, and even lead to difficulties in nail placement. Obesity will increase the difficulty of retrograde placement of traditional pubic branch screws and the risk of vital organ puncture injury (32–34). In our study, the screw entry point is located in the larger gap between the femoral blood vessel and the spermatic cord, which is far away from the perineum compared with the traditional retrograde pubic branch screw, and the screw placement is safer. Although obesity also increased the difficulty of pinning in our experience, we have not encountered a case of failure due to obesity factor yet, but it should be noted. Due to the single plane fixation of the screw, patients with pelvic ring fractures who have both rotational and vertical instability are prone to screw loosening (35). Therefore, single plane rotational instability of TileB type fracture is the best indication for this technique. For those with a small diameter of the pubic branch and a small angle between the pubic symphysis from the entrance view, the lack of linear screw channels would to some extent limit the application of this technology. By pre bending the pin tip, changing the direction of the intramedullary canal, and cutting the affected cortex with screws, the above limitations could be avoided to some extent.

Generally speaking, as a supplement to minimally invasive treatment of pelvic fractures, this study might be a good choice for patients who are unable to receive open surgery or cannot tolerate prolonged bed rest. As the shortcoming of the study, the number of cases in this study is yet relatively small and it is not a controlled study, although the efficacy observed so far is encouraging. In addition, this study mainly introduce the fixation methods and nail placement techniques of symphysis pubis fracture, and related closed reduction techniques are not involved in this paper, which could expand the scope of use of this technology.

## Data Availability

The original contributions presented in the study are included in the article/Supplementary Material, further inquiries can be directed to the corresponding author.
